# Trophoblast Differentiation Affects Crucial Nutritive Functions of Placental Membrane Transporters

**DOI:** 10.3389/fcell.2022.820286

**Published:** 2022-02-22

**Authors:** Rona Karahoda, Jonas Zaugg, Barbara Fuenzalida, Sampada Kallol, Ruedi Moser-Haessig, Frantisek Staud, Christiane Albrecht

**Affiliations:** ^1^ Department of Pharmacology and Toxicology, Faculty of Pharmacy in Hradec Kralove, Charles University, Hradec Kralove, Czech Republic; ^2^ Institute of Biochemistry and Molecular Medicine, University of Bern, Bern, Switzerland; ^3^ Swiss National Centre of Competence in Research, NCCR TransCure, University of Bern, Bern, Switzerland; ^4^ Division of Gynecology and Obstetrics, Lindenhofgruppe, Bern, Switzerland

**Keywords:** trophoblast, placental barrier, membrane transport, nutrients, fetal programming, pregnancy pathology, cell differentiation

## Abstract

Cytotrophoblasts are progenitor cells that proliferate and fuse to form the multinucleated syncytiotrophoblast layer, implicated in placental endocrine and transport functions. While membrane transporters play a critical role in the distribution of nutrients, hormones, and xenobiotics at the maternal-fetal interface, their selectivity to the syncytiotrophoblast layer is poorly characterized. We aimed to evaluate the regulation of placental transporters in response to trophoblast differentiation *in vitro*. Experiments were carried out in isolated primary human trophoblast cells before and after syncytialization. Gene expression of six molecular markers and thirty membrane transporters was investigated by qPCR analysis. Subsequently, functional expression was evaluated for proteins involved in the transplacental transfer of essential nutrients i.e., cholesterol (ABCA1, ABCG1), glucose (SLC2A1), leucine (SLC3A2, SLC7A5), and iron (transferrin receptor, TfR1). We identified that human chorionic gonadotropin, placental lactogen, endoglin, and cadherin-11 serve as optimal gene markers for the syncytialization process. We showed that trophoblast differentiation was associated with differential gene expression (mostly up-regulation) of several nutrient and drug transporters. Further, we revealed enhanced protein expression and activity of ABCG1, SLC3A2, SLC7A5, and TfR1 in syncytialized cells, with ABCA1 and GLUT1 displaying no change. Taken together, these results indicate that the syncytiotrophoblast has a dominant role in transporting essential nutrients cholesterol, leucine, and iron. Nonetheless, we present evidence that the cytotrophoblast cells may also be linked to transport functions that could be critical for the cell fusion processes. Our findings collectively yield new insights into the cellular functions associated with or altered by the trophoblast fusion. Importantly, defective syncytialization could lead to nutrient transfer imbalance, ultimately compromising fetal development and programming.

## 1 Introduction

The placenta is the first and largest fetal organ created during human development. In the first weeks of pregnancy, trophectoderm, the precursor of placental cells, interacts with the uterine epithelium allowing implantation (Burton and Fowden 2015). This process leads to the generation of mononucleated cytotrophoblast cells (CTBs), precursors of extravillous and villous trophoblasts. In the anchoring villus, CTBs subsequently differentiate by fusion to the multinucleated syncytiotrophoblast (STB) layer, representing the primary maternal-fetal exchange site. These complex processes are highly orchestrated, involving biochemical and morphological changes to support the integrity of the placental exchange surface (Knofler et al., 2019). Importantly, as a highly endocrine organ, the secretion of hormones and proteins can serve as markers to distinguish the placental cellular source and play a role in deciphering processes associated with trophoblast differentiation. Nonetheless, despite the high importance, this field of research is still in its infancy, mainly due to the lack of suitable cellular models (Knofler et al., 2019).

With the fetus entirely dependent on the maternal supply of nutrients, the placental nutrient exchange is critical for proper fetal development and programming (Costa 2016). The placenta is equipped with a battery of transporters that facilitate the transfer of nutrients and control the transplacental disposition of many drugs (Vähäkangas and Myllynen 2009; Koepsell 2020). Two families of membrane transport proteins are recognized: the ATP-binding cassette (ABC) and the solute carrier (SLC) transporter family. The ABC transporters actively pump their substrates out of the trophoblast cells into the maternal circulation, using ATP as an energy source; thus, they play a critical role in fetal protection against drugs and other toxins (Dallmann et al., 2019). Additionally, ABC transporters orchestrate the biodistribution of several endogenous substrates, including steroid hormones, lipids, cholesterol, and factors involved in inflammatory and immunological responses (Bloise et al., 2016). SLC transporters mainly facilitate the transport of charged and uncharged organic molecules as well as inorganic ions into the trophoblast cells and they are involved in materno-fetal nutrient translocation and metabolic sensing (Walker et al., 2017; Zhang et al., 2018). Several members of this diverse transporter family are functionally expressed in the trophoblast cells, mediating the transport of amino acids, glucose, monoamines, organic cations and anions, and a wide range of drugs and toxins (Balkovetz et al., 1989; Illsley 2000; Sata et al., 2005; Cleal et al., 2018).

Altered placental transport function represents one of the critical mechanisms implicated with Developmental Origins of Health and Disease (Barker et al., 1993; Barker et al., 2010). Maternal under- or over-nutrition elicits metabolic changes, which have a profound regulatory effect on placental nutrient transport. These adaptive mechanisms subsequently increase the offspring’s risk of metabolic and cardiovascular disturbances in adulthood (Gaccioli et al., 2013). Additionally, impaired placental transporters are implicated in placental pathologies, including gestational diabetes mellitus, pre-eclampsia, and fetal growth restriction (Desoye et al., 2011; Huang et al., 2018; Zaugg et al., 2020b).

Although several ABC and SLC transporters have been described in the placenta (for summary see Table 1), limited evidence still exists on their localization, developmental changes, and the extent of functionality within the fetoplacental unit. Understanding the expression patterns and functional activity of placental transporters is essential in providing insights into their role in reproductive functions and pregnancy-associated pathologies. Emerging evidence highlights that placental transporters may participate in intracellular signaling and cell differentiation (Apáti et al., 2016; Bloise et al., 2016). Nonetheless, while there is a general concept that the STB represents the main transport surface in the fetoplacental unit, only a limited number of studies have investigated the transport capacities of trophoblast cells in relation to their differentiation state (Evseenko et al., 2006; Kallol et al., 2018a; Kallol et al., 2018b; Zaugg et al., 2020a).

**TABLE 1:** Overview of key placental ABC and SLC transport systems with associated genes and gene products, evaluated in the study.

Transporter family	Gene name	Protein name	Aliases	Transport type	Substrates	References
Cholesterol efflux transporter	ABCA1	ABCA1	ABC1, TGD, CERP	A	cholesterol, phospholipids,	Kallol and Albrecht (2020)
	ABCG1	ABCG1	ABC8	A	cholesterol	
Drug transporter	ABCB1	P-gp	ABC20, CD243, MDR1	A	steroids, bilirubin, bile acids, anticancer drugs, protease inhibitors, drugs of abuse	Vähäkangas et al. (2011)
	ABCG2	BCRP	ABCP, BCRP1, MXR	A	estrones, bile acids, glyburide, cimetidine, statins, anticancer drugs	
Facilitative glucose transporter	SLC2A1	GLUT1		F	glucose, galactose, mannose, glucosamine	Illsley (2000)
Folate transporter	SLC19A1	RFC	RFC1, RFT	E/organic phosphates	reduced folates, antifolates	Zhao and Goldman (2013)
Thiamine transporter	SLC19A2	THTR1	ThTr1	F	thiamine	
	SLC19A3	THTR2	ThTr2	F	thiamine	
Proton-coupled metal ion transporter	SLC11A2	DMT1	NRAMP2, DCT1	C/H^+^	Fe^2+^, Cd^2+^, Co^2+^, Cu^1+^, Mn^2+^, Ni^2+^, Pb^2+^, Zn^2+^	Montalbetti et al. (2013)
Metal ion transporter	SLC39A8	ZIP8, BIGM103, LZT-Hs6			Zn, Cd, Mn	Jeong and Eide (2013)
	SLC39A14	ZIP14, LZT-Hs4			Zn, Fe, Mn, Cd	
	SLC40A1	FPN1	MTP1, IREG1	F?	Fe^2+^	Montalbetti et al. (2013)
Proton oligopeptide co-transporter	SLC15A1	PEPT1	oligopeptide transporter 1, H^+^-peptide transporter 1	C/H^+^	di- and tri-peptides, protons, beta-lactam antibiotics	Smith et al. (2013)
	SLC15A2	PEPT2	oligopeptide transporter 2, H^+^-peptide transporter 2	C/H^+^	di- and tri-peptides, protons, beta-lactam antibiotics	
Transporters for anionic amino acids	SLC1A2	GLT-1, EAAT2	System X^−^_AG_	C/Na^+^, H^+^, K^+^	Glu, Asp	Kanai et al. (2013)
	SLC1A3	GLAST, EAAT1	System X^−^_AG_	C/Na^+^, H^+^, K^+^	Glu, Asp	
	SLC7A11	xCT	[4F2hc], system xc^-^	E (Cys against Glu)	cystine (anionic form), glutamate	Fotiadis et al. (2013)
Na^+^-dependent transporters for neutral amino acids	SLC38A1	SNAT1	ATA1, NAT2, SAT1	C/Na^+^	Gln, Ala, Asn, Cys, His, Ser	Schiöth et al. (2013)
	SLC38A2	SNAT2	ATA2, SAT2	C/Na^+^	Ala, Asn, Cys, Gln, Gly, His, Met, Pro, Ser	
Na^+^-independent transporters for neutral amino acids	SLC7A5	LAT1	[4F2hc], 4F2lc, system L	E	large neutral amino acids, triiodothyronine (T3), thyroxine (T4), DOPA, BCH	Fotiadis et al. (2013), Scalise et al. (2018)
	SLC7A8	LAT2	[4F2hc], system L	E	neutral amino acids, T3, T4, BCH	
	SLC43A1	LAT3	POV1	F	branched chain amino acids, amino alcohols	Bodoy et al. (2013)
	SLC43A2	LAT4		F	branched chain amino acids, amino alcohols	
Cationic amino acids and large neutral L-amino acids transporter	SLC7A1	CAT-1	ATRC1, system y^+^	F (non-obligatory E)	cationic L-amino acids	Fotiadis et al. (2013)
	SLC7A6	y^+^LAT2	[4F2hc], system y^+^L	E	cationic amino acids (Na^+^ independent), large neutral amino acids (Na^+^ dependent)	
	SLC7A7	y^+^LAT1	[4F2hc], system y^+^L	E	cationic amino acids (Na^+^ independent), large neutral L-amino acids (Na^+^ dependent)	
	SLC7A9	b^0,+^AT	[rBAT], system b^0,+^	E	cationic amino acids, large neutral amino acids	
Heavy subunits of the heteromeric amino acid transporters (SLC7)	SLC3A1	rBAT	NBAT, D2H	E	system b^0,+^, heterodimerizes with light subunit SLC7A9	Fotiadis et al. (2013), Napolitano et al. (2015)
	SLC3A2	4F2hc	CD98hc, FRP	E	systems L, y^+^L, xc^−^ and asc with light subunits SLC7A5-8 and SLC7A10-11	

Abbreviations for transport type: A: Active; C: Co-transporter; E: Exchanger; F: Facilitated transporter. The information on SLC transporters was constructed partially based on the online resource for solute carriers Bioparadigms (www.bioparadigms.org).

In this context, we sought to determine the expression and functional activity of several membrane transport proteins in primary CTBs isolated from the human term placenta. When seeded on extracellular matrix-coated dishes, CTBs spontaneously fuse over time into multinuclear STB (Kliman et al., 1986), thus providing an ideal physiological model to study processes associated with cell differentiation. Several molecular markers have been suggested to identify villous trophoblast cells, such as cytokeratin, ERVW-1, hCG, CDH11, ENG, MUC1, and hPL (Huppertz 2006). While immunohistochemistry is often used for this purpose, we sought to determine the mRNA expression of selected markers to observe whether their expression reflects the differentiation stage *in vitro*. Next, we screened for differential expression of several classes of nutrient and drug transport genes associated with trophoblast syncytialization. The selection of transporters (Table 1) was based on experimental evidence that these proteins influence the transplacental transfer of essential nutrients (e.g., glucose, lipids, amino acids), micronutrients (e.g., iron), and vitamins (e.g., folate, thiamine) that are of crucial importance for placental and fetal development and metabolic programming. Lastly, we investigated the functional regulation of membrane proteins that transport representative substrates of selected nutrient classes (i.e., cholesterol, leucine, and iron), thus providing insights into the transport functions associated with or altered by the trophoblast fusion process.

## 2 Materials and Methods

### 2.1 Tissue Collection

Human term placenta samples (gestational age at delivery: 38–40) were collected from uncomplicated pregnancies after elective cesarean section at the Division of Gynecology and Obstetrics, Lindenhofgruppe, Bern, Switzerland. All women signed informed consent, and the study was performed according to the Declaration of Helsinki, with the approval of the Ethics Committee of the Canton of Bern (Basec Nr. 2016-00250).

### 2.2 Primary Trophoblast Isolation, Characterization, and Culture

Villous trophoblast cells were isolated using a previously established protocol from human term placenta samples (Kliman et al., 1986; Kallol et al., 2018a). Briefly, villous tissue was subjected to three enzymatic digestion steps at 37°C with 0.25% trypsin (Sigma, United States) and 300 IU/ml deoxyribonuclease I (Sigma, United States). The obtained cell suspension was overlayed on fetal bovine serum (Seraglob, Switzerland), centrifuged, and the pellet was resuspended on Dulbecco’s Modified Eagle’s Medium (high glucose; Gibco, United Kingdom). Subsequently, a discontinuous Percoll^®^ gradient was used to separate the cells. The purity of the isolated cells was evaluated by staining for cytokeratin-7 (93% positivity), vimentin (4% positivity), and E-cadherin (75% positivity) using flow cytometry as previously described (Kallol et al., 2018a). Collected cells were seeded in CellBIND^®^ plates (Costar, United States) and cultured in Dulbecco’s Modified Eagle Medium (high glucose) with 10% FBS and 1% antibiotic–antimitotic. CTB stage is represented by cells cultured for 8 h after seeding, whereas the STB stage is obtained by spontaneous fusion of CTBs over 72 h of culturing (with daily change of medium). hCG secretion by CTB and STB was measured in the cell culture medium using the human hCG (intact) ELISA kit (Sigma, United States), following the manufacturer’s instructions.

### 2.3 Quantitative PCR Analysis

Total RNA was isolated from primary human trophoblast cells (CTB and STB stage) using Trizol reagent (Invitrogen, United Kingdom). RNA concentration and purity were determined spectrophotometrically using the NanoDrop 1000 Spectrophotometer (Thermo Fisher Scientific, United States). Subsequently, RNA was reverse transcribed to cDNA with the GoScript™ Reverse Transcriptase System (Promega, United States) according to the manufacturer’s instructions. PCR analysis was carried out using the ViiA™7 RT-PCR System (Applied Biosystems, United States) and the SYBR^®^ Green PCR master mix detection kit (Promega, United States), as previously described (Kallol et al., 2018b). The primers used are listed in Supplementary Table S1. The mRNA expression of target genes was normalized against the expression of tyrosine 3-monooxygenase/tryptophan 5-monooxygenase activation protein, zeta polypeptide (YWHAZ), stably expressed during trophoblast differentiation as previously described (Kallol et al., 2018b). Subsequently, relative expression to the undifferentiated cells was calculated and the results are expressed as 2^−ΔΔCt^ or as median log_2_ Fold Change (FC).

### 2.4 Protein Expression Analysis by Western Blot

Cells (CTB and STB stage) were lysed in protein extraction buffer (100 mmol/L NaCl, 0.5% Triton X-100, 1% SDS, 50 mmol/L Tris/HCl, pH 7.4) containing a mixture of protease inhibitors. Extracts were sonicated, and the protein content was determined using the Pierce BCA Assay Kit (Thermo Fisher Scientific, United States). 50–70 µg of proteins were separated as described (Fuenzalida et al., 2018) by polyacrylamide gel electrophoresis (8%), transferred to nitrocellulose membrane (Protran BA-83; Sigma-Aldrich, United States), and probed with primary rabbit polyclonal anti-ABCA1 (1:500, 18 h, 4°C; Novus Biological, United States), anti-ABCG1 (1:500, 18 h, 4°C; 1945-1, Epitomics, United States), anti-SLC7A5/LAT1 (1:500, 18 h, 4°C; 85226, Abcam, United Kingdom), anti SLC3A2/4F2hc (1:1000, 18 h, 4°C; sc-376815, Santa Cruz Biotechnology, United States), anti-SLC2A1/GLUT1 (1:1000, 18 h, 4°C; 07-1401, Millipore, United States) and anti-CD71/TfR1 (1:1000, 18 h, 4°C; GTX102596, GeneTex, United States). The corresponding signal was visualized after incubation with anti-rabbit/anti-mouse IgG secondary antibody (1:20,000, 2 h, RT; 926-68071/926-32210, LI-COR, United States) by chemiluminescence (ODYSSEY Imaging System, LI-COR Biosciences, United States) and normalized to 0.1% (w/v) Ponceau S in 5% acetic acid (v/v) staining (P7170, Sigma). Ponceau S-stained blots can be found in Supplementary Figure S1.

### 2.5 Lipoprotein Isolation

For efflux assays, lipoproteins from non-pregnant donors were isolated by ultracentrifugation as described (Sreckovic et al., 2013). Briefly, sucrose (final concentration: 10%), EDTA (10 mmol/L, pH 7.4), aprotinin (2 μg/ml) and phenylmethylsulfonyl fluoride (1 mmol/L) were added to serum. The serum density was adjusted with KBr to 1.24 g/ml. PBS (1.006 g/ml) was added over the samples to generate a density gradient by ultracentrifugation (rotor SW55Ti, 287 000 RCF, 15°C, 4 h), from which LDL, HDL, and the lipoprotein-depleted serum (LPDS) were isolated. After dialysis in saline solution (150 mmol/L NaCl, 0.34 mmol/L EDTA, pH 7.4, 4°C, 48 h), LDL and HDL were stored at 4°C in a sealed tube saturated with nitrogen. The protein concentration was determined using the Pierce BCA Protein Assay Kit (Thermo Fisher Scientific, United States). The isolation efficiency was determined by SDS-PAGE separation followed by Coomassie R-250 staining and western blot analysis for ApoB and ApoA-I.

### 2.6 Cholesterol Efflux Assays

The efflux of [^3^H]-cholesterol from trophoblast cells to ApoA-I or HDL was determined with minor modifications as described (Kallol et al., 2018a; Fuenzalida et al., 2020). In brief, cells were seeded at a density of 0.3 × 10^6^ cells/cm^2^ in 24-well plates and allowed to attach for 8 h for CTB and 48 h for STB. Then cells were pre-incubated for 24 h with Dulbecco’s Modified Eagle Medium (high glucose) containing 10% FBS supplemented with [^3^H]-cholesterol (0.5 μCi/ml). Subsequently, cells were washed with PBS supplemented with BSA-FFA (2 mg/ml). Next, cells were incubated at 37°C for 6 h with non-pregnant isolated HDL (50 μg/ml) or ApoA-I (10 μg/ml; Sigma-Aldrich, United States). Finally, the culture medium was recovered, and the cells were lysed with KOH. The efflux activity was estimated as the fraction of radioactive signal in the medium compared to the total signal in the medium and cells. Acceptor-mediated efflux is calculated by subtracting efflux without acceptor from efflux with acceptor.

### 2.7 Leucine Uptake

Leucine uptake was determined based on a published method (Häfliger et al., 2018), as previously described (Zaugg et al., 2021). Primary trophoblast cells were incubated in Na^+^-free Hank’s buffer (125 mM choline chloride, 25 mM HEPES, 4.8 mM KCl, 1.2 mM MgSO_4_, 1.2 mM KH_2_PO_4_, 1.3 mM CaCl_2_, 5.6 mM glucose, pH 7.4) containing 1 μCi/ml [^3^H]-L-[3,4,5-3H(N)]-leucine (PerkinElmer, United States) and unlabeled L-leucine at a final concentration of 167.2 μmol/L. Leucine uptake was stopped by three washing steps, and cell lysis was achieved by adding the scintillation solution (MicroScint-20, PerkinElmer, United States) and plate shaking for 90 min at room temperature. Finally, radioactivity was determined using the TopCount^®^ NXT™ Scintillation and Luminescence Counter (PerkinElmer, United States).

### 2.8 Transferrin-Mediated Iron Uptake

The experimental procedure and analysis of transferrin-mediated iron uptake were adapted from Gambling et al. (2001), as previously described (Zaugg et al., 2020b). In brief, ^55^FeCl_3_ (PerkinElmer, Germany)-labeled uptake solution was prepared by dissolving human Apo-Tf in a balanced salt solution as previously reported (Zaugg et al., 2020b). Tf binding was carried out at 37°C for 2 h. Subsequently, the cells were washed and equilibrated for 30 min at 37°C before the diluted uptake solution was added. The final iron concentration was 179.8 nmol/L. Iron uptake was stopped at defined time points, and cell lysis was achieved by adding the scintillation solution (MicroScint-20, PerkinElmer, United States) and plate shaking for 90 min at room temperature. Radioactivity was measured by TopCount^®^ NXT™ Scintillation and Luminescence Counter (PerkinElmer, United States), and iron uptake was calculated as previously described (Zaugg et al., 2020b).

### 2.9 Statistical Evaluation and Graphical Presentation

Data analysis and graphical presentation were performed using the GraphPad Prism^®^ software (GraphPad, United States). Expression and functional data were evaluated using nonparametric *t*-tests (Mann-Whitney test), whereas Two-Way ANOVA assessed time-dependent leucine and iron uptake.

## 3 Results

### 3.1 Expression and Secretion of Syncytial Markers

Initially, mononucleated CTBs and the multinucleated STB layer (differentiated *in vitro* over a 72-h culture period) were analyzed for the expression of reported markers indicative of the syncytialization process (Huppertz 2006). Specifically, the expression of human chorionic gonadotropin (hCG), human placental lactogen (hPL), cadherin 11 (CDH11), endoglin (ENG), mucin-1 (MUC1), and syncytin-1 (ERVW-1) was investigated (Table 2). As expected, we observed a significant upregulation in hCG, hPL, and ENG gene expression at the STB stage compared to CTBs. Likewise, hCG protein release at 72 h was significantly higher in the differentiated cells, confirming the physiological characteristics of our *in vitro* model. On the other hand, while CDH11 showed a tendency towards higher expression in STB (*p* = 0.052), MUC1 and ERVW-1 were transcriptionally similar in both cell states.

**TABLE 2:** Evaluation of mRNA expression and protein secretion of commonly used syncytialization markers.

Marker	log_2_^FC^	*p*-value
hCG mRNA	8.87 (5.86–9.98)	*p* = 0.0005
hCG secretion (ng/ml)	1.87 (0.98–2.22)	*p* = 0.002
hPL mRNA	1.60 (1.06–2.75)	*p* = 0.0034
CDH11 mRNA	1.49 (0.11–3.57)	*p* = 0.052
ENG mRNA	2.26 (0.63–4.11)	*p* = 0.0068
MUC1 mRNA	-1.38 (-2.24–0.41)	*p* = 0.19
ERVW-1 mRNA	-0.081 (-0.69–0.83)	*p* > 0.99

Gene expression of human chorionic gonadotropin (hCG), human placental lactogen (hPL), cadherin 11 (CDH11), endoglin (ENG), mucin-1 (MUC1), and syncytin-1 (ERVW-1) was evaluated by qPCR analysis and normalized to the expression of YWHAZ. hCG, protein secretion was measured using an ELISA kit. Presented results are log_2_^FC^, expression/secretion in STB stage compared to the CTB counterpart. Data are shown as median with IQR; n ≥ 10. Statistical significance was evaluated using nonparametric *t*-test (Mann-Whitney test).

### 3.2 Effect of Trophoblast Differentiation on the Gene Expression of Membrane Transporters

**FIGURE 1:**
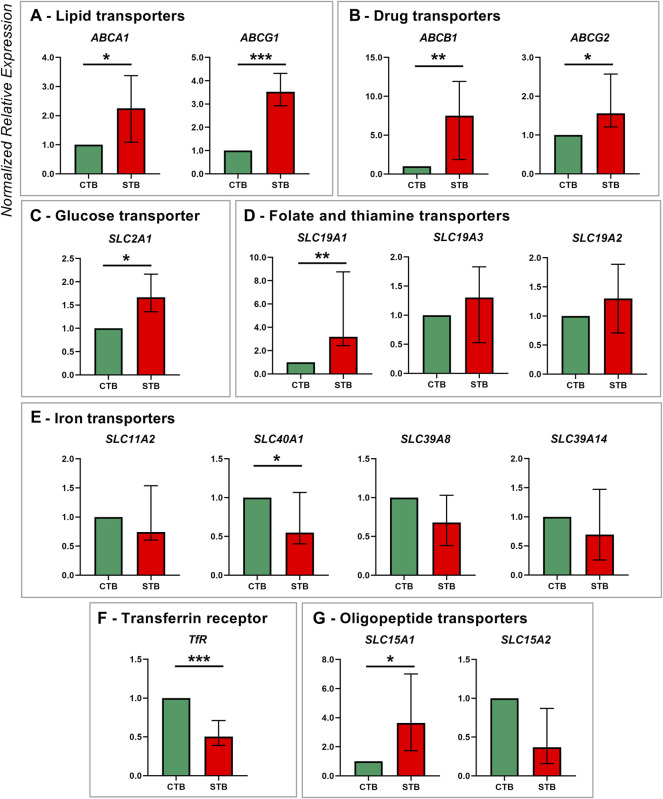
Membrane transporter gene expression in human term primary CTBs and upon spontaneous differentiation to STB *in vitro*. mRNA expression was evaluated by qPCR analysis for members of the following transport classes: lipids **(A)**, drugs **(B)**, glucose **(C)**, folate and thiamine **(D)**, iron **(E)**, and peptide **(G)**; additionally, expression of the iron uptake receptor was assessed **(F)**. Target gene expression was normalized to the reference gene YWHAZ and the results are shown as median with IQR expression in differentiated cells relative to the undifferentiated counterpart; *n* ≥ 7 for each stage. Statistical significance was evaluated using nonparametric t-tests (Mann-Whitney test): *p ≤ 0.05, **p ≤ 0.01, ***p ≤ 0.001.

**FIGURE 2:**
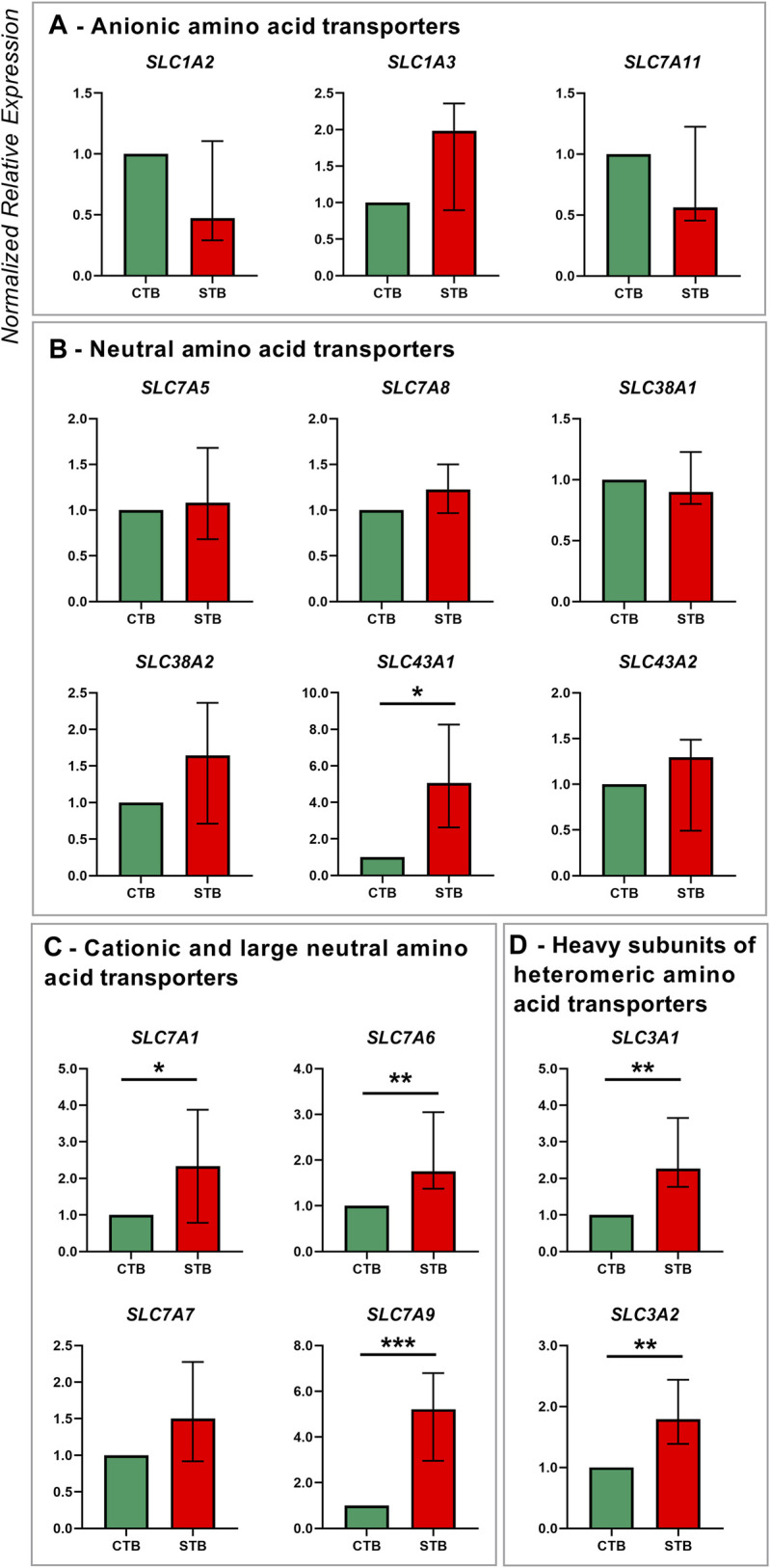
Amino acid transporter gene expression in human term primary CTBs and upon spontaneous differentiation to STB *in vitro*. Classification of amino acid transporters was based on the substrate specificity: anionic **(A)**, neutral **(B)**, cationic and large neutral **(C)**, and heavy subunits of the heterodimeric amino acid transporters **(D)**. Target gene expression was normalized to the reference gene YWHAZ and the results are shown as median with IQR expression in differentiated cells relative to the undifferentiated counterpart; *n* ≥ 7 for each stage. Statistical significance was evaluated using nonparametric *t*-tests (Mann-Whitney test): *p ≤ 0.05, **p ≤ 0.01, ***p ≤ 0.001.

Thirty transporters of the ABC and SLC superfamily were evaluated for their expression patterns in CTB and STB cells (Figures 1,2). Concerning lipid transporters (Figure 1A), ABCA1 and ABCG1 were selected as primary transporters mediating cholesterol efflux from cells to lipid-poor ApoA-I and HDL particles, respectively (Kallol and Albrecht 2020). The expression of both transporters was significantly upregulated in the differentiated cells. Similarly, the expression of two other efflux transporters from the ABC superfamily, namely ABCG2 and ABCB1, facilitating the transport of several substrates (including drugs and environmental chemicals (Vähäkangas and Myllynen 2009)), was significantly upregulated in the STB stage (Figure 1B).

Several members of the SLC superfamily were examined, particularly those mediating the transport of glucose, iron, vitamins, peptides, and amino acids. mRNA expression of glucose transporter SLC2A1 was significantly upregulated in the differentiated trophoblast cells (Figure 1C). Of the SLC19A gene family, only SLC19A1 (mediating the transport of reduced folate) showed higher expression in STB. In contrast, thiamine transporters SLC19A2 and SLC19A3 were similarly expressed in CTB and STB cells (Figure 1D). While placental iron transport mechanisms are still not fully elucidated, we investigated the expression of five key proteins reported in the literature (Sangkhae and Nemeth 2019), comprising four transporters of the SLC superfamily (SLC11A2, SLC40A1, SLC39A8, SLC39A14; Figure 1E) and transferrin receptor (TfR1; Figure 1F). Thereof SLC40A1 (Figure 1E) and TfR1 (Figure 1F) showed differential expression patterns, with higher expression in CTBs and downregulated expression in the STB stage. Additionally, the expression of peptide transporters (SLC15A family) was analyzed, revealing significant upregulation of the SLC15A1 transporter at the STB stage, but no change for SLC15A2 (Figure 1G).

Regarding amino acid transporters, five families from the SLC superfamily were examined and they include members of the SLC1A, SLC3A, SLC7A, SLC38A, and SLC43A families (Figure 2). The STB cell stage was mainly associated with an upregulation of transporters for cationic and large neutral amino acid transporters SLC7A1, SLC7A6, and SLC7A9 (Figure 2C). Similarly, the expression of heavy subunits of heteromeric amino acid transporters SLC3A1 and SLC3A2 was significantly increased in the differentiated cells (Figure 2D). On the other hand, the expression of transporters for anionic (SLC1A2, SLC1A3, SLC7A11; Figure 2A), and neutral amino acids (SLC7A5, SLC7A8, SLC38A1, SLC38A2, SLC43A2; Figure 2B), was unaltered by the spontaneous differentiation process. The only exception was the Na^+^-independent transporter for neutral amino acids SLC43A1, which was upregulated in STB cells (Figure 2B).

**FIGURE 3:**
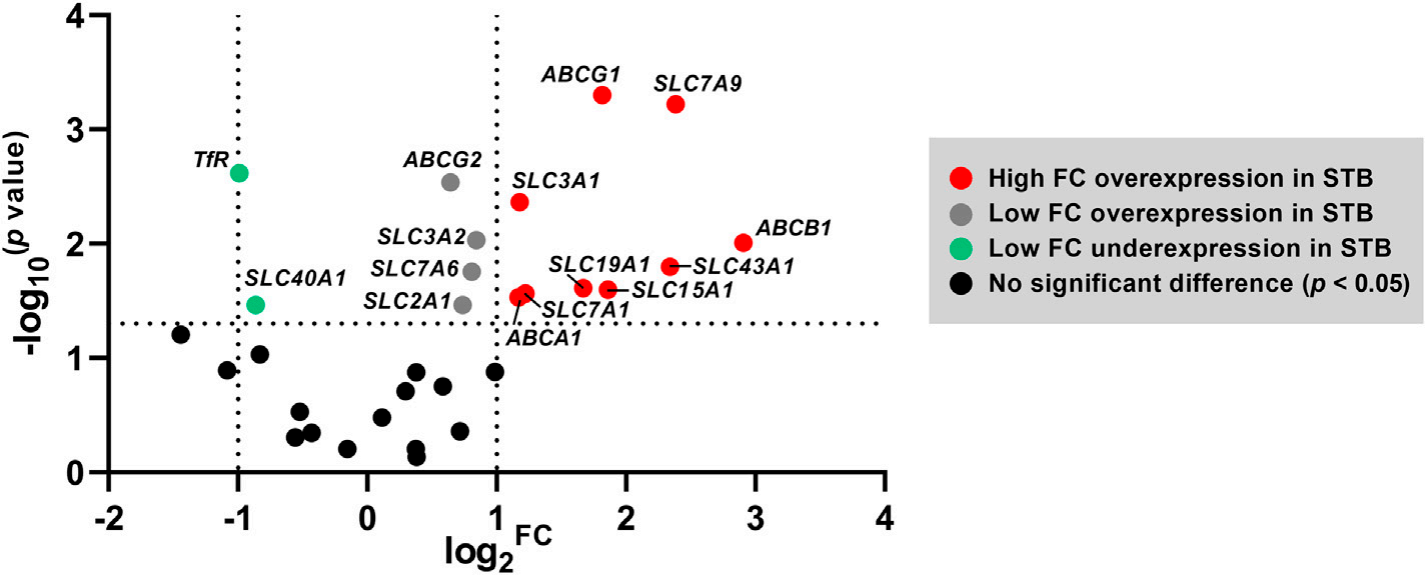
Volcano plot highlighting the set of genes that are differentially expressed at the STB stage. The horizontal axis represents median log_2_ fold change (FC), and the vertical axis −log_10_ transformed *p* values; statistical analysis was evaluated by nonparametric t-tests (Mann-Whitney test). The dotted lines represent the upper and lower limit of the threshold FC (which was set to 2) and the threshold *p*-value (set as < 0.05).

Collectively, trophoblast differentiation under physiological conditions *in vitro* is predominantly characterized by an overexpression of several membrane transport proteins. Figure 3 further highlights the genes with the highest fold-change overexpression in STB (FC > 2) comprising cholesterol efflux transporters (ABCA1, ABCG1), the drug transporter ABCB1, the oligopeptide transporter SLC15A1, the folate transporter SLC19A1, and several amino acid transporters (SLC3A1, SLC7A1, SLC7A9, SLC43A1).

### 3.3 Protein Expression of Selected Membrane Transporters in Primary Trophoblast Cells

**FIGURE 4:**
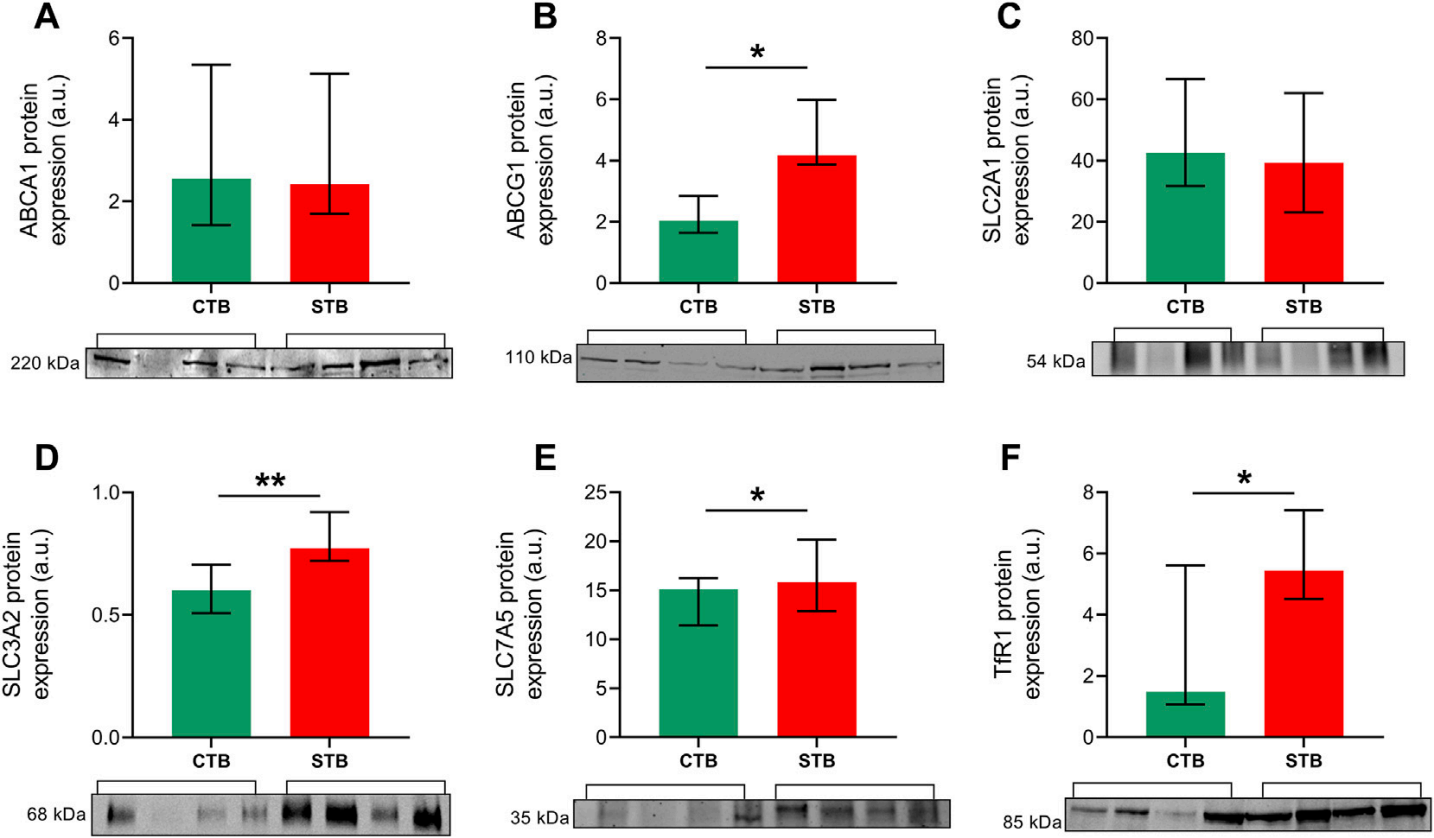
Expression of selected proteins of interest in primary trophoblast cells. Western blot analysis was carried out to evaluate the protein expression of cholesterol efflux transporters ABCA1 **(A)** and ABCG1 **(B)**, glucose uptake transporter SLC2A1 **(C)**, 4F2 heavy chain SLC3A2 **(D)** and amino acid transporter SLC7A5 **(E)**, and iron uptake receptor TfR1 **(F)**. Protein expression was normalized to the total protein, as visualized by Ponceau S staining. Data are shown as median with IQR; *n* = 4 for each stage. Asterisks indicate significance according to nonparametric *t*-test (Mann-Whitney test): *p ≤ 0.05, **p ≤ 0.01.

Western blot analysis was performed to confirm whether the differential mRNA expression is also reflected at the protein level for representative genes of nutrient transport proteins. Cell lysates from the CTB and STB stage were incubated with antibodies encoding ABCA1, ABCG1, SLC2A1, SLC3A2, SLC7A5, and TfR1. Protein expression of ABCG1 (Figure 4B) and SLC3A2 (Figure 4D) was consistent with the findings from gene expression studies, showing significantly higher expression in the differentiated cell state. Contrary to mRNA levels, SLC7A5 (Figure 4E) and TfR1 (Figure 4F) protein expression was significantly upregulated upon trophoblast differentiation *in vitro*. On the other hand, ABCA1 (Figure 4A) and SLC2A1 (Figure 4C) protein expression seemed to be unaffected by the differentiation stage.

### 3.4 Effect of Trophoblast Differentiation on Cholesterol Efflux and Leucine and Iron Uptake

**FIGURE 5:**
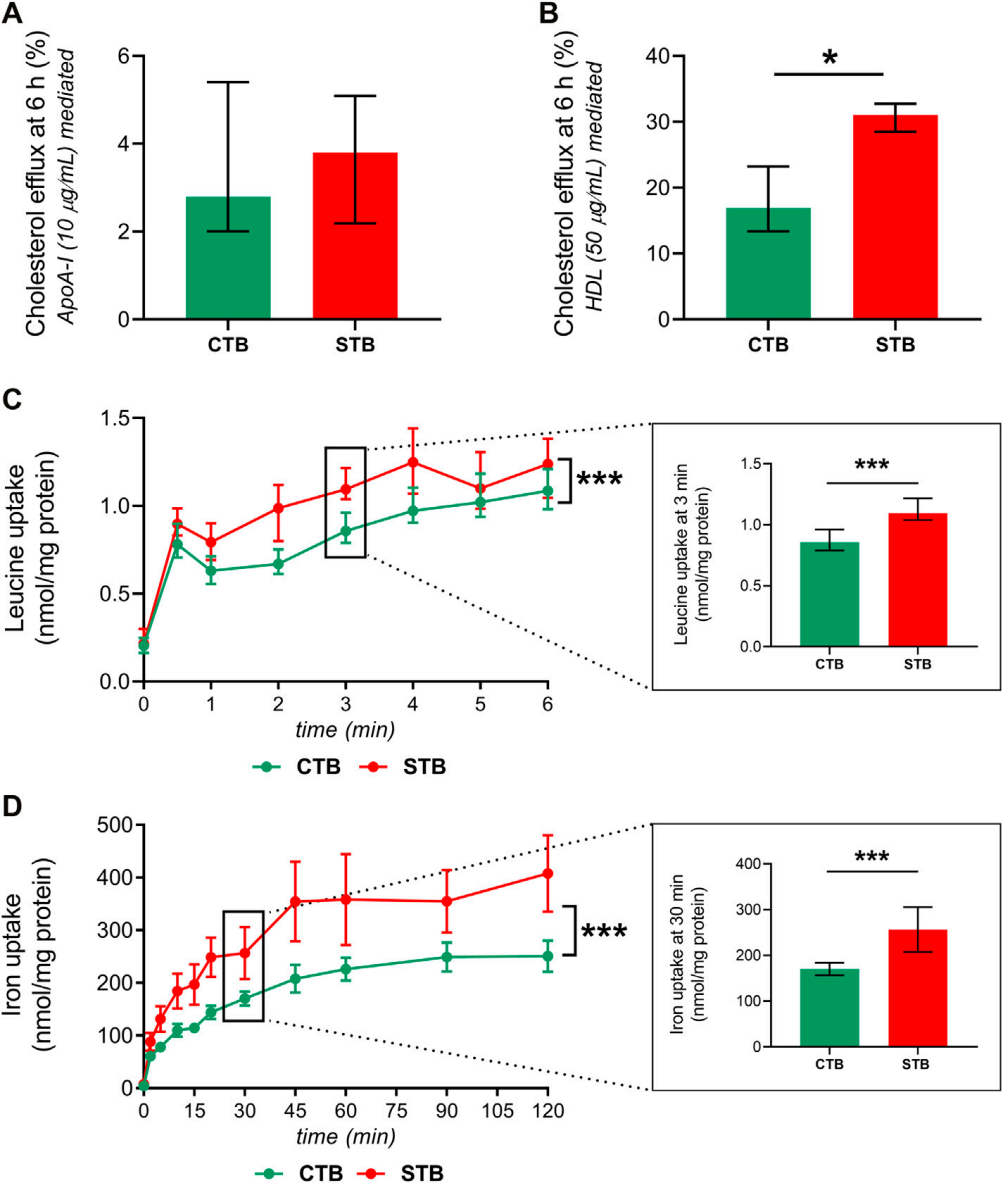
Effect of trophoblast differentiation on the transport of cholesterol, leucine, and iron. CTB, 8 h, and STB, 48 h after seeding, were incubated for 24 h with [^3^H]-cholesterol (0.5 μCi/ml). Subsequently, cholesterol efflux was evaluated in the presence of 10 μg/ml ApoA-I **(A)**, ABCA1-mediated) and 50 μg/ml HDL **(B)**, ABCG1-mediated) for 6 h. The efflux transporter activity is shown as the fraction of radioactive signal in the medium compared to the total signal in the medium and cells. Acceptor-mediated efflux is calculated by subtracting efflux without acceptor from efflux with the acceptor. Leucine **(C)**, SLC7A5-mediated) and iron **(D)**, TfR1-mediated) uptake in CTB and STB was assessed in a time-dependent manner, and the results are shown in nmol/mg protein. Data are presented as median with IQR; *n* ≥ 3 for each stage. Asterisks indicate significance according to paired, nonparametric *t*-test (Wilcoxon test) or two-way ANOVA (for time-dependency studies): *p ≤ 0.05, ***p ≤ 0.001.

To investigate functional differences in the transporter activity between the CTB and STB stage, three representative essential substrates from the class of lipids (cholesterol), amino acids (leucine), and micronutrient (iron) transporters were selected. The efflux activity of ABCA1 and ABCG1 was determined by analyzing cholesterol transport in the presence of ApoA-I and HDL, respectively. At a 6-h incubation period, we observed higher ABCG1 (HDL-mediated) efflux activity in the STB stage (Figure 5B). On the other hand, ABCA1-mediated cholesterol to ApoA-I was not affected by the differentiation process (Figure 5A). Leucine and iron uptake was investigated in a time-dependent manner, revealing increased uptake over time in both CTB and STB cells; nonetheless, the uptake in the STB stage was significantly higher than CTB cells (Figures 5C,D).

## 4 Discussion

**FIGURE 6:**
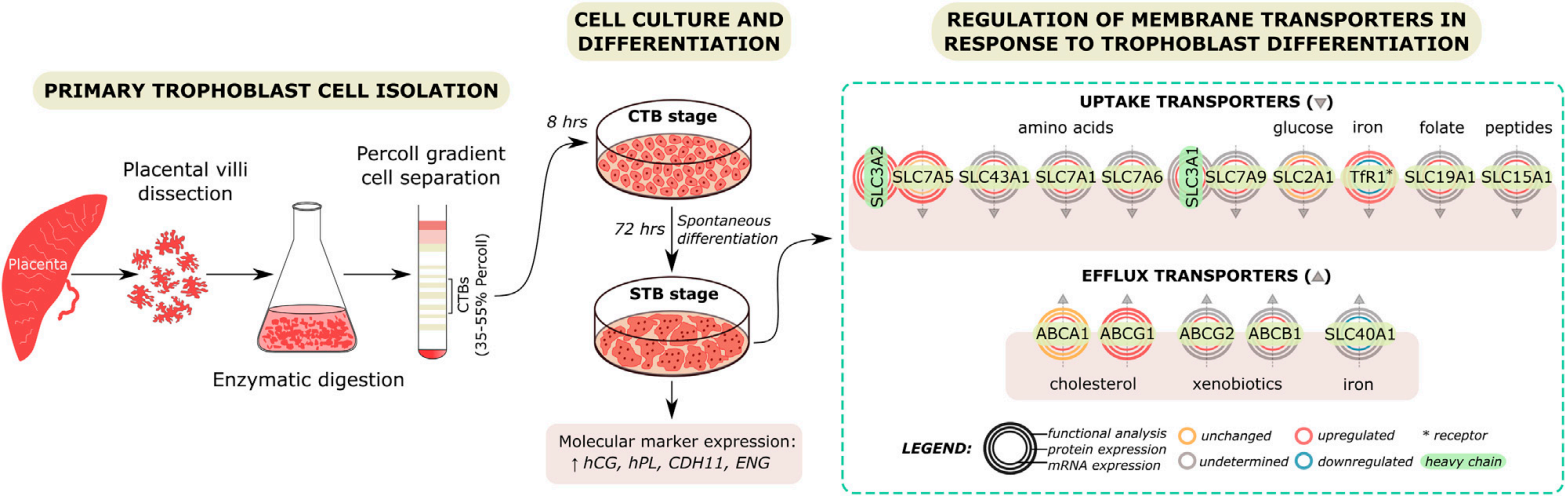
Regulation of membrane transport protein expression and function by trophoblast differentiation. Primary trophoblast cells were isolated from the human term placenta. Dissected tissue was subjected to enzymatic digestion and Percoll gradient cell separation. Isolated cytotrophoblast (CTB) cells were cultured *in vitro* and allowed to spontaneously differentiate to syncytiotrophoblast (STB) over time. Experiments were carried out at a 8- or 72-h culture period, representing the CTB and STB stages, respectively. Molecular marker expression and secretion were used to confirm that cell differentiation was achieved. Subsequently, the effect of trophoblast differentiation on the expression and function of placental membrane proteins highly relevant for fetal development was evaluated. Our findings reveal differential membrane transport protein function between the CTB and STB stage, likely reflecting their involvement in the differentiation and transport functions in individual cells.

Hormone synthesis and transplacental nutrient delivery comprise principal functions for optimal fetal growth and development (Costa 2016). The STB layer is the crucial structure implicated with maternal-fetal exchange due to its critical position and high metabolic rate (Carter 2000). On the contrary, the latest research has shown that the undifferentiated CTBs are the most metabolically active cells in the human term placenta, with a high fuel flexibility level (Kolahi et al., 2017). Deciphering the mechanisms which govern and/or are affected by trophoblast differentiation is crucial in the fundamental understanding of placental biology. This study provides a comprehensive evaluation of several endocrine and transport aspects associated with the differentiation of CTBs into STB (Figure 6).

Using qPCR analysis, we show that the STB stage is associated with upregulated gene expression of hCG, hPL, ENG, and CDH11. Upregulated hCG gene expression was further confirmed by hCG protein release in the cell supernatant. This is in line with reported literature using immunohistochemical analysis (MacCalman et al., 1996; Frank et al., 2001; Pötgens et al., 2003) and demonstrates the physiological characteristics of our *in vitro* model. Further, this finding identifies mRNA expression as an excellent means of determining the spontaneous syncytialization process of CTB *in vitro*. On the other hand, we report similar mRNA expression levels of MUC1 and ERVW-1 in freshly isolated CTBs and the multinucleated STB. MUC1 expression was previously described in both villous trophoblast cell populations (Jeschke et al., 2002); herein, we report that its mRNA expression is unaffected by CTB fusion. ERVW-1 expression in CTB cells, however, is not well described. Its expression has mainly been related to the STB layer, a phenomenon that appears paradoxical considering ERVW-1’s *in vitro* cell-cell fusogenic activity (Pötgens et al., 2004). To this end, using RNA-Seq gene expression, Azar et al. recently reported down-regulated ERVW-1 in the STB cell stage (Azar et al., 2018). Importantly, ERVW-1 knockout mice show defective syncytial formation and placentation, overexpansion of CTBs, and die at midgestation (Dupressoir et al., 2009). Taken together, these findings and our data suggest that ERVW-1 is consistently expressed throughout the differentiation processes, and in the CTB stage, it plays an important role in cell fusion processes.

We have previously conveyed the expression of several membrane transporters in the BeWo cell line (before and after syncytialization with forskolin) and primary trophoblast cells (CTB and STB stage) (Kallol et al., 2018b). In the current study, we concentrate on primary trophoblast cells isolated from the human term placenta and investigate in detail the expression signature of thirty membrane nutrient transport proteins (Table 1) in a significantly larger dataset. We show that the STB stage is associated with upregulated gene expression of cholesterol efflux transporters. Cholesterol is an essential membrane component, a precursor of steroid hormones, and plays a crucial role in embryogenesis (Kallol and Albrecht 2020). Despite fetal cholesterol synthesizing capacity, maternal cholesterol remains a critical source for the fetus, and thus, maternal-to-fetal transport is essential (Woollett 2011). Maternal cholesterol is carried by lipoproteins such as LDL or HDL, taken up by respective receptors. Subsequently, cholesterol efflux from the cell is active and is mediated by ABCA1 and ABCG1 onto lipid-poor ApoA-I and HDL, respectively (Kallol and Albrecht 2020). We provide evidence that the STB cells display upregulated ABCA1 and ABCG1 gene expression, whereas only for ABCG1, this change is reflected in the protein level. These expression patterns were further accompanied by differential cholesterol efflux capacity.

Specifically, we show that ABCG1-mediated cholesterol efflux in STB (promoted by HDL) is significantly enhanced, whereas ABCA1-mediated transport (promoted by ApoA-I) is unaffected by the differentiation stage. Our observations are in line with the findings by Keelan et al., who showed that after a 72-h culture time (also used in our study), ABCA1 mRNA expression is significantly upregulated in the STB stage, whereas protein expression is unaltered (Keelan et al., 2011); nonetheless, the authors further report significantly increased ABCA1 protein after a 120-h incubation period. It should be noted that cholesterol efflux is also significant during CTB differentiation, and ABCA1-deficient mice show significant placental malformations (Christiansen-Weber et al., 2000). Interestingly, ApoA-I release by primary trophoblast cells is also the highest at 24 h of culture (CTB stage) (Melhem et al., 2019). This suggests higher ApoA-I availability in the CTB cell phase, likely promoting ABCA1-mediated cholesterol efflux for processes within the undifferentiated cell. Indeed studies have shown that, in addition to transmembrane transport, ABCA1 also moves lipids within membranes to modulate cell proliferation and immunity (Nagao et al., 2011). Additionally, ABCA1 upregulation modifies cholesterol packaging in cell membranes, altering plasma membrane properties (Hassan et al., 2007), which may contribute to its potential involvement in cell fusion processes (Bocchetta et al., 2014).

The trophoblast syncytialization process was recently associated with the enrichment of genes involved in amino acid transport (Azar et al., 2018). We show that this primarily involves key transporters for cationic and large neutral amino acids (SLC7A1, SLC7A6, and SLC7A9), while the expression of anionic and neutral amino acid transporters is not significantly affected. Interestingly, we also observed that the heavy subunits for the heterodimeric amino acid transporters, SLC3A1 and SLC3A2, are significantly upregulated during the *in vitro* trophoblast differentiation. The only amino acid transporter known to form heterodimer with SLC3A1 (also named rBAT) is SLC7A9 (b^0,+^AT) (Fotiadis et al., 2013). While the rBAT/b^0,+^AT complex has been reported in the placenta (Feliubadaló et al., 1999), its functional impact remains undetermined. Nonetheless, the parallel increase in SLC3A1 and SLC7A9 mRNA expression during trophoblast differentiation suggests the potential importance of this complex in the STB stage and thus requires further investigation. On the other hand, SLC3A2 (also named 4F2hc) forms heterodimers with a broader range of transporters from the SLC7A family, i.e., the neutral amino acid transporters (SLC7A5, SLC7A8), cationic and large neutral amino acid transporters (SLC7A6, SLC7A7), and the anionic amino acid transporter SLC7A11 (Fotiadis et al., 2013). In addition to its role in targeting these transporters, SLC3A2 has also been reported to exert cellular fusogenic and proliferative action (Cantor et al., 2009; Takesono et al., 2012). Recent work in trophoblast cells has highlighted the importance of SLC7A5 in supporting protein expression and membrane presentation of SLC3A2 for fusogenic activity (Ohgaki et al., 2017). To further understand the complex relationship between trophoblast differentiation and SLC7A5/3A2, we investigated their protein and functional expression in CTB and STB cells. In line with our previous reports (Zaugg et al., 2020a), we show that the expression of these proteins and uptake of leucine (SLC7A5 substrate) is significantly enhanced in STB compared to the CTB cells. These findings suggest that trophoblast differentiation is associated with a parallel upregulated transport capacity. Collectively, this phenomenon, previously also reported for other amino acid transport systems (Furesz et al., 1993), highlights the dominant role of STB in amino acid transfer across the placental barrier.

To support the fetal nutrient demands, pregnancy profoundly affects maternal physiology and metabolism, including iron homeostasis (Sangkhae and Nemeth 2019). Optimal iron nutrition is essential for fetal development and the establishment of iron stores upon delivery. The major maternal iron source is iron complexed with transferrin, whose uptake is mediated by transferrin receptor 1 (TfR1). Several other transport proteins (including SLC11A2, SLC40A1, SLC39A8, SLC39A14) are reportedly highly expressed in the placenta contributing to the maternal-to-fetal transfer of iron (Sangkhae and Nemeth 2019). Nonetheless, despite their vital importance, our understanding of iron transporter localization to the trophoblast cells remains limited. Here, we show that all investigated iron transport proteins are expressed in both CTB and STB cells. Interestingly, TfR1 mRNA expression showed downregulation upon trophoblast differentiation *in vitro*. To examine this further, protein expression and functional analysis of transferrin-mediated iron uptake were measured. Contrary to mRNA level, CTB differentiation to STB provoked expressional and functional upregulation in TfR1 protein. As TfR1 represents a key player in iron transport, these findings suggest that the STB is the principal placental cell mediating transplacental iron delivery to the fetus.

In conclusion, this study yields new insights into the cellular functions associated with or altered by trophoblast fusion. Quantitative PCR analysis was applied as a screening method for differential mRNA expression of several classes of nutrient and drug transport genes associated with trophoblast syncytialization. However, we were limited in addressing protein and functional alterations of all transporters investigated. As such, it is the first step in understanding the physiological functions linking placental transport function with trophoblast differentiation. The abnormal trophoblast differentiation process has been proposed as a potential mechanism underlying the pathophysiology of several placental pathologies (Huppertz 2011; Szilagyi et al., 2020). Defective syncytialization could lead to nutrient transfer imbalance, ultimately compromising fetal development and programming. Therefore, our findings are relevant to several studies indicating altered placental transport in pregnancy-associated conditions (Desoye et al., 2011; Huang et al., 2018; Zaugg et al., 2020b). Importantly, with a large number of transporters currently reported in the human placenta, future studies should focus on deciphering the expression patterns in trophoblast cells and their involvement in cellular functions. Knowledge of this nature may potentially facilitate the development of treatment strategies in pregnancy complications associated with these transport proteins.

## Data Availability

The original contributions presented in the study are included in the article/Supplementary Material, further inquiries can be directed to the corresponding author.
