# Supplementary material for: Trophoblast Differentiation Affects Crucial Nutritive Functions of Placental Membrane Transporters
Source: Front Cell Dev Biol. 2022 Feb 22;10:820286. doi: 10.3389/fcell.2022.820286 (PMC8901483; doi:10.3389/fcell.2022.820286)
Supplement: Supplementary file 1 [file DataSheet1.docx]

Supplementary Material

**Supplementary Table 1.** Primer Characteristics.

| Gene | Primer sequence (5’ end to 3’ end) | Fragment length (bp) |
| --- | --- | --- |
| *ABCA1* | for: CCACATTTTTGCCTGGGACG  rev: AGCGATTCTCCCCAAACCTT | 88 |
| *ABCG1* | for: AACATGGAGGCCACTGAGAC  rev: GGCCACCAACTCACCACTAT | 228 |
| *ABCB1* | for: GCCAGAAACAACGCATTGCC  rev: GGGCTTCTTGGACAACCTTTTC | 117 |
| *ABCG2* | for: GAGGCAAATCTTCGTTAT  rev: CCATCACAACATCATCTT | 114 |
| *SLC1A2* | for: GGAAGAAGAATGATGAAGTG  rev: ACTGTTTGAATCTGTTGAAA | 106 |
| *SLC1A3* | for: GTAGCAGTAATAATGTGGTATG  rev: TGAGTAAGCCAACAATGA | 136 |
| *SLC2A1* | for: GAACTCTTCAGCCAGGGTCC  rev: ACCACACAGTTGCTCCACAT | 114 |
| *SLC3A1* | for: CCTTCAACAATTACCTCAG  rev: CCACCAATCATCCAGTTA | 118 |
| *SLC3A2* | for: AGCTGGAGTTTGTCTCAGGC  rev: GGCCAATCTCATCCCCGTAG | 127 |
| *SLC7A1* | for: CTGGCAGCTCACGGAGGAGGA  rev: GCCCCCGACAGGACACCAGA | 129 |
| *SLC7A5* | for: CAGGGCATCTTCTCCACGAC  rev: TGGGTTCGAGGAGGTGATCTA | 137 |
| *SLC7A6* | for: CCGAGGCAGACAAGTGGAAT  rev: TGTTTGCTGTGGCCTGTCAA | 131 |
| *SLC7A7* | for: AGGCATTTGTCTGGCCTTCCCTTT  rev: TGGCATTGCCTCCTTGGTCCTG | 144 |
| *SLC7A8* | for: AGGCCCTCCTCTGTGGCTGG  rev: GTGCAGCAGGAGTGGCTGGG | 106 |
| *SLC7A9* | for: AAACCCTTACAGAAACCT  rev: AAGCAGGATAGAGAACAC | 167 |
| *SLC7A11* | for: TGCTGGGCTGATTTATCTTCG  rev: GAAAGGGCAACCATGAAGAGG | 114 |
| *SLC11A2* | for: TTGCGGAGCTGGTAAGAATCA  rev: AAGACTGGCAGACTCCCCAT | 126 |
| *SLC15A1* | for: GAGCAATCCTGATTCTGTA  rev: ACACAATGGTCTTGAACT | 156 |
| *SLC15A2* | for: TGTCTGCTTATAGAACTGT  rev: CCTGATTGGTGTTATTAGTAAT | 141 |
| *SLC19A1* | for: CGAGTCGCAGGCACAGC  rev: CACGGGCACCTGCTTCTC | 101 |
| *SLC19A2* | for: TCACGATAGCAACTTTTC  rev: GGCATCTACCACAATTAG | 125 |
| *SLC19A3* | for: ACCAGTGCAGAGATAACAA  rev: ACATAATCGGTGAGGACAA | 92 |
| *SLC38A1* | for: GCATACTCTTGGTTGTTATC  rev: GATGTAACTCCTACGACTC | 82 |
| *SLC38A2* | for: GACTTCAACTACTCCTACC  rev: ACCTGGATGAAATTCTGT | 135 |
| *SLC39A8* | for: GACAGTTATGTTGAGAAGG  rev: TGACCATTCTGACCATAT | 107 |
| *SLC39A14* | for: CTGCTGCTCTACTTCATA  rev: CAGACTTGGAGACATAATAATC | 118 |
| *SLC40A1* | for: AGATCACAACCGCCAGAGAG  rev: CACATCCGATCTCCCCAAGT | 111 |
| *SLC43A1* | for: CCTGCCTTATCTTTCTGA  rev: CTTCTTCGTGTAATTGACTT | 83 |
| *SLC43A2* | for: TTCCTCAACTGCTTCTTTA  rev: GATCTTCACCGAGTAGTC | 75 |
| *TfR1* | for: GTAGATGGCGATAACAGT  rev: CCAATCATAAATCCAATCAAGA | 167 |
| *YWHAZ* | for: CCGTTACTTGGCTGAGGTTG  rev: AGTTAAGGGCCAGACCCAGT | 143 |
| *hCG* | for: CGGGACATGGGCATCCAA  rev: GCGCACATCGCGGTAGTT | 202 |
| *hPL* | for: GCTATGCTCCAAGCCCAT  rev: TGCAGGAATGAATACTTCTGGT | 101 |
| *CDH11* | for: TCTCCTGGTCATTGTAGTAT  rev: TTCTCACGGACATCTTCT | 90 |
| *ENG* | for: CAAGACCAGGAAGTCCATA  rev: CGTGTGCGAGTAGATGTA | 174 |
| *MUC1* | for: CACCGACTACTACCAAGA  rev: GCACATCACTCACTGAAC | 111 |
| *ERVW-1* | for: GATATTTGGCTAAGGAGGTGATGTC  rev: GAAGGCCCTTCATAACCAATGA | 83 |


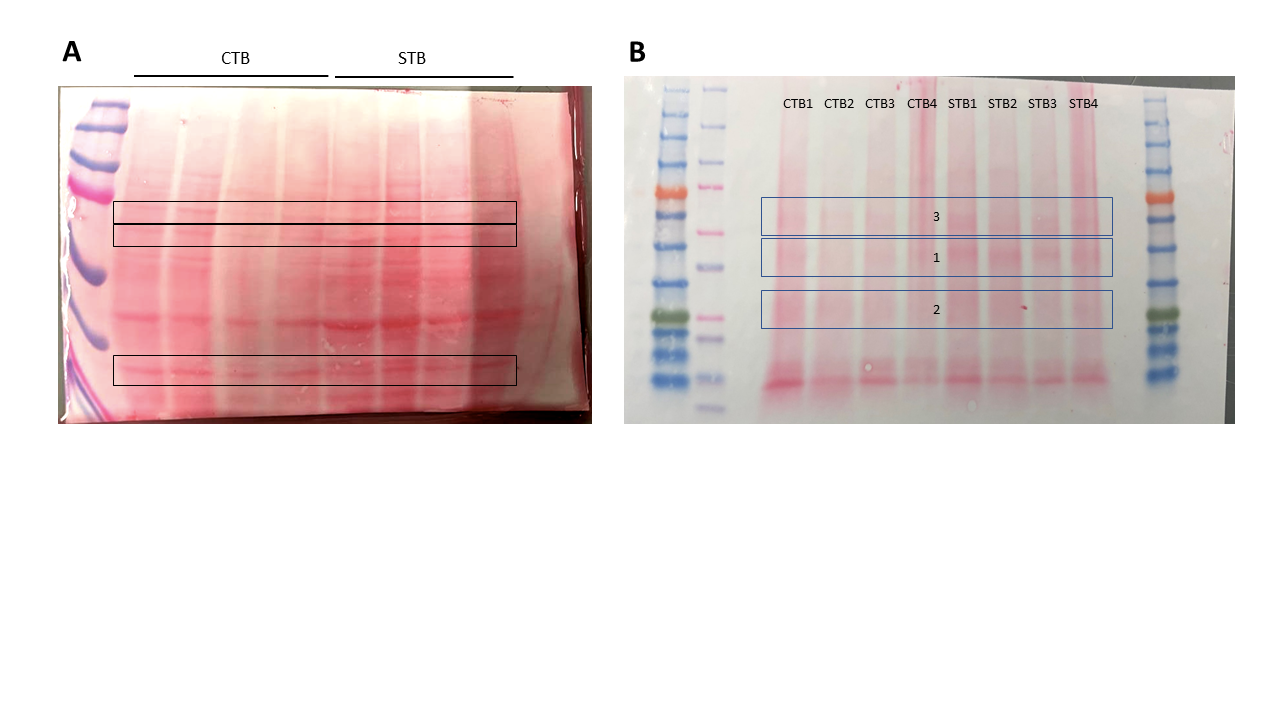


**Supplementary Figure 1.** Ponceau S-stained blots for A) ABCA1, ABCG1, TfR1, and B) SLC3A2, SLC7A5, SLC2A1. 50-70 µg of proteins were separated by polyacrylamide gel electrophoresis (8%), transferred to nitrocellulose membrane, and stained with Ponceau S stain for normalization.
